# Rescue procedure for left main bronchus obstruction after patent ductus arteriosus clipping: a case report

**DOI:** 10.1186/s40792-018-0471-y

**Published:** 2018-06-28

**Authors:** Yukiko Ban, Masakazu Abe

**Affiliations:** Department of Cardiovascular Surgery, Ibaraki Children’s Hospital, 3-3-1, Futabadai, Mito, Ibaraki 311-4145 Japan

**Keywords:** Patent ductus arteriosus, Surgical complication, Airway obstruction

## Abstract

**Background:**

Obstruction of the left main bronchus is a rare but life-threatening complication following the closure of patent ductus arteriosus by surgical clips. We report a successful rescue procedure for this complication in a premature infant.

**Case presentation:**

A 24-week gestational age premature girl weighing 903 g underwent surgical clipping for patent ductus arteriosus at the age of 24 days after birth. Bronchoscopy revealed the left main bronchus obstruction due to the clip compression at 6 h later after the surgery. The patient underwent a rescue re-exploration for this serious complication. New clips were applied to both the intrapericardial and the aortic ends of the patent ductus arteriosus respectively. And then the previous clips, compressing the left main bronchus, were gently removed from the ductus without ductus injury through a re-thoracotomy.

**Conclusions:**

Surgeons should be aware of the possible complication and take care not to place patent ductus arteriosus clips obliquely toward the bronchus.

## Background

Patent ductus arteriosus (PDA) is a relatively common and harmful condition in premature infants. PDA injury, recurrent laryngeal, or phrenic nerve injuries are well-recognized complications in surgical closure of PDA. Obstruction of the left main bronchus is a rare complication following PDA closure with surgical clips. Because PDA is located close to the left main bronchus, inappropriate placement in the size, position, depth, and/or angle of the clips may cause serious airway obstruction. We report a case of obstruction of the left main bronchus following PDA closure with surgical clips and a successful rescue procedure for this life-threatening complication without PDA injury.

## Case presentation

**Fig. 1:**
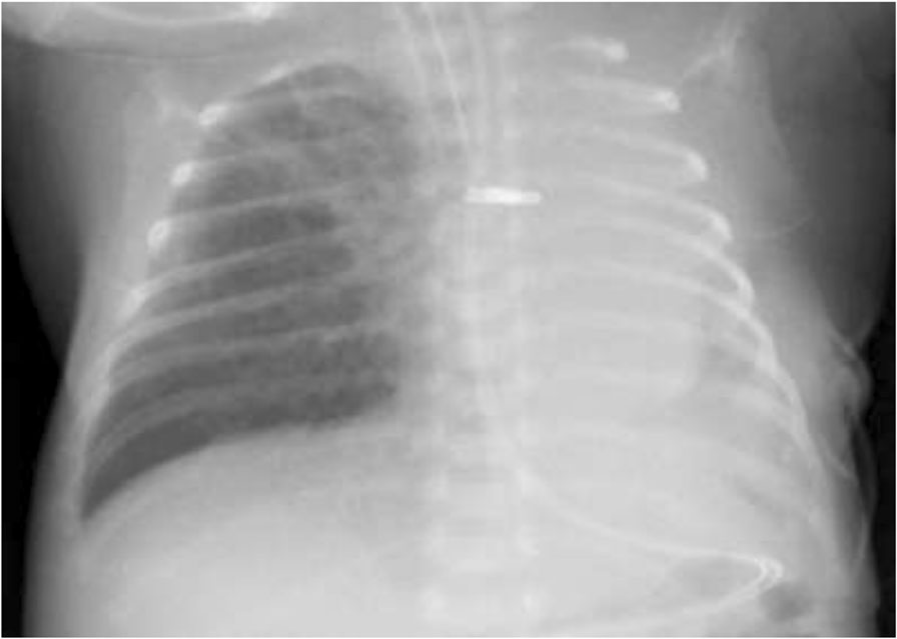
Chest X-ray after PDA ligation showed completely collapsed left lung

**Fig. 2:**
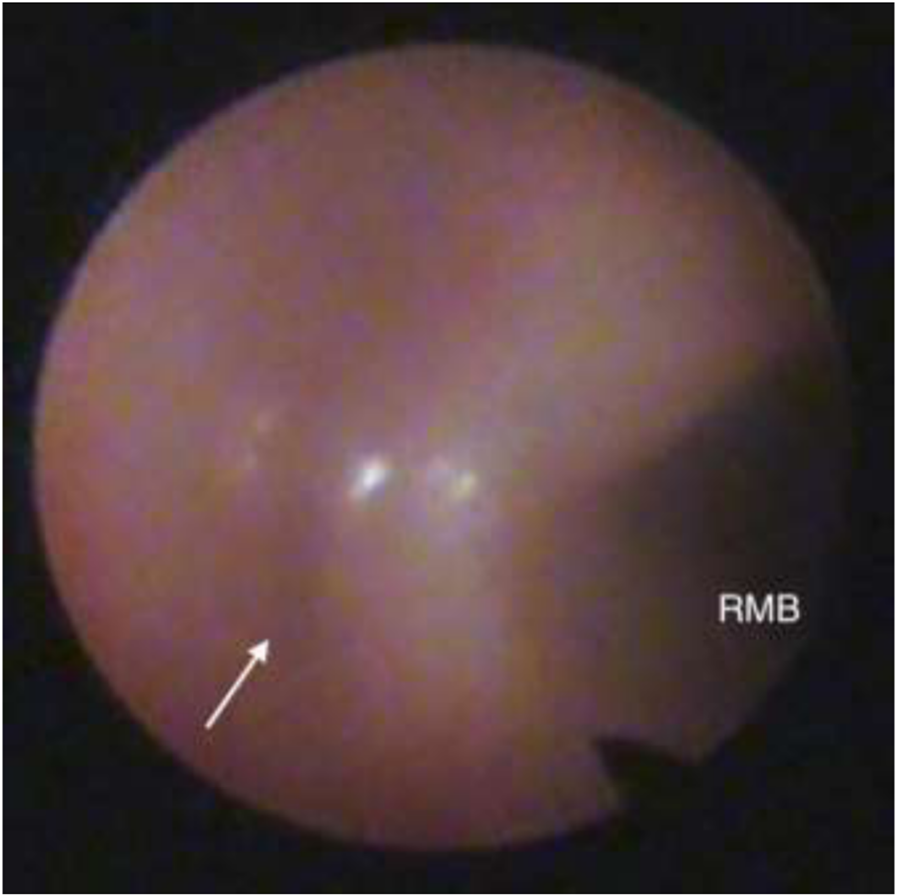
Bronchoscopy showed a complete obstruction of the left main bronchus (white arrow) (RMB = right main bronchus)

A 24-week gestational age premature girl weighing 903 g underwent surgical intervention for PDA at the age of 24 days after birth. The PDA was 3 mm in diameter and closed with two medium-sized, 4.5-mm-long surgical clips through a left thoracotomy. Although we recognized atelectasis of the left lung after the PDA clipping, it was improved by the adjustment of the airway pressure and tracheal tube position except for a part of the left lower lobe. However, chest X-ray film after the procedure still showed atelectasis of the left lung (Fig. 1). In spite of meticulous respiratory treatments, left atelectasis did not improve at all. The patient developed critical desaturation 6 h after surgery and required high airway pressure for ventilation. Bronchoscopy revealed that the left main bronchus was occluded completely by the external compression of the PDA clips at the lateral wall (Fig. 2). The patient immediately underwent a rescue re-exploration for this serious complication.

**Fig. 3:**
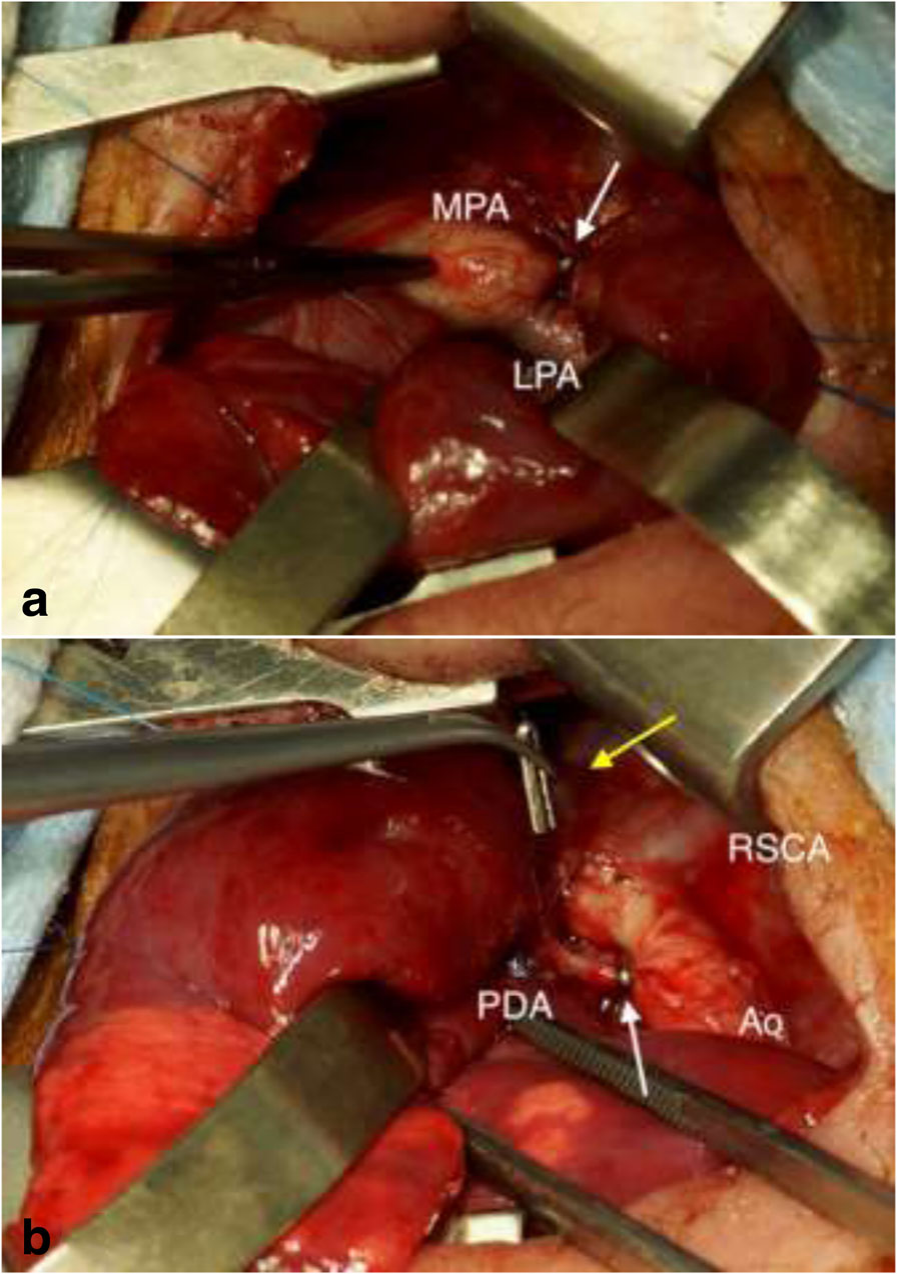
Surgical procedure. **a** PDA was clipped at the proximal end through a pericardiotomy (white arrow). **b** Another new clip was applied on the aortic end (white arrow), and the previous two clips were safely removed (yellow arrow) (Ao = aorta; LPA = left pulmonary artery; MPA = main pulmonary artery; PDA = patent ductus arteriosus; RSCA = right subclavian artery)

At re-exploration, one of the tips of the clips was tilted anteriorly and caudally and appeared to be applied obliquely toward the carina of the trachea. First, we prepared that the aorta could be temporarily clamped above and below the PDA. We applied two new clips on both ends of the PDA; one clip was placed on the intrapericardial pulmonary end of the PDA through a pericardiotomy, and another clip was placed on the aortic end (Fig. 3a, b). Subsequently, the previously applied two PDA clips were carefully removed from the PDA (Fig. 3b). This procedure was accomplished without PDA or airway injuries. Atelectasis of the left lung immediately improved after the rescue procedure. The postoperative course was uneventful, and the patient was extubated 3 days later.

### Discussion

Obstruction of the left main bronchus is a rare but life-threatening complication of PDA closure with surgical clips. Harris et al. [1] reported that the PDA clip had been placed across the left main bronchus and caused the left main bronchus obstruction. A significant compression of the left main bronchus by a surrounding fibrous mass was noted, and they needed to remove the mass and skeletonize the left main bronchus 2 months after the PDA surgery. Bhat et al. [2] reported that a single PDA clip was applied to both the PDA and the left main bronchus, and they needed to repair the left main bronchus injury 5 days after the PDA surgery. In these cases, chest computed tomography showed the clip applied across the left main bronchus.

In our case, the possibility that the PDA clip was applied across the left main bronchus was presumed to be low, considering the clip size and PDA diameter. We believe that closing the PDA on both ends of the PDA in advance would prevent hemorrhage in case of PDA injury during clip removal. Approaching through a pericardiotomy, the new clip on the pulmonary end was placed away from the carina and did not cause airway obstruction. Extensive dissection of the clipped PDA was hazardous; therefore, we clipped PDA again with minimal dissection. If new clips could not be applied both ends of the PDA, aortic and/or left pulmonary artery clamp might be useful.

In the present case, an inappropriate placement angle of the clips resulted in the airway obstruction. The left main bronchus is located close beneath to the PDA and has a markedly thin wall of fragile cartilages in premature infants. A rigid metal clip placed inappropriately on the PDA compresses and occludes the fragile airway easily. Surgeons should apply PDA clips carefully after considering their position, angle, and depth to avoid airway complications. In order to clip PDA without airway obstruction, surgeons should hold a clip vertically and apply it to the extreme aortic end of PDA.

It is important not to hesitate to perform re-exploration in case of suspected airway obstruction by PDA clips to prevent a deleterious outcome. Both previously documented reports suggest that the surgical rescue procedure may become complicated if the re-exploration is performed late after the initial operation. An early bronchoscopic detection of the complication is necessary to prevent a more complicated reoperation. Surgeons, therefore, should be aware of this possible complication.

## Conclusions

We described left main bronchus obstruction after patent ductus arteriosus clipping. Surgeons should be aware of this possible complication and take care not to place the PDA clips toward the left main bronchus.

## Abbreviation

PDA: Patent ductus arteriosus

## References

[1] Harris LL, Krishnamurthy R, Browne LP, Morales DL, Friedman EM (2012). Left main bronchus obstruction after patent ductus arteriosus ligation: an unusual complication. Int J Pediatr Otorhinolaryngol.

[2] Bhat AN, John J, Riyas MK, AlKurdi B, Salama H (2014). Inclusion of the left main bronchus in the clip used to occlude the ductus arteriosus in a premature baby: an unexpected complication. Indian J Anaesth.

